# Effects of compression clothing on speed–power performance of elite Paralympic sprinters: a pilot study

**DOI:** 10.1186/s40064-016-2681-8

**Published:** 2016-07-11

**Authors:** Irineu Loturco, Ciro Winckler, Thiago F. Lourenço, Amaury Veríssimo, Ronaldo Kobal, Katia Kitamura, Lucas A. Pereira, Fábio Y. Nakamura

**Affiliations:** NAR - Nucleus of High Performance in Sport, Av. Padre José Maria, 555 - Santo Amaro, São Paulo, SP 04753-060 Brazil; CPB – Brazilian Paralympic Committee, Brasília, DF Brazil; UEL – State University of Londrina, Londrina, PR Brazil

**Keywords:** Sprint, Squat jump, Visual impairment, Blind athletes, Loaded jumps

## Abstract

**Background:**

Compression garments are thought to aid performance in some selected speed–power activities owing to improved sensory feedback and proprioception. The aim of this study was to test the effects of using compression garments on speed and power-related performances in elite sprinters with visual impairment, who rely more on proprioception to perform than their Olympic peers. Eight top-level Paralympic sprinters competing in 100- and 200-m races performed, in the following order: unloaded squat jump (SJ), loaded jump squat (JS) and sprint tests over 20- and 70-m distances; using or not the compression garment. The maximum mean propulsive power value obtained during the JS attempts (starting at 40 % of their body mass, after which a load of 10 % of body mass was progressively added) was considered for data analysis purposes. The athletes executed the SJ and JS attempts without any help from their guides. Magnitude-based inference was used to analyze the results.

**Findings:**

The unloaded SJ was *possibly higher* in the compression than the placebo condition (41.19 ± 5.09 vs. 39.49 ± 5.75 cm). Performance differences in the loaded JS and sprint tests were all rated as *unclear*.

**Conclusions:**

It was concluded that the acute enhancement in vertical jump ability should be explored in the preparation of Paralympic sprinters during power-related training sessions. However, chronic effects in Paralympic athletes wearing compression garments need to be further tested, in order to support its use as a specific training aid.

## Background

 In elite sports, coaches often seek for acute strategies to optimize training performance in order to obtain competitive benefits. For instance, traditional warm-up and post-activation potentiation strategies have been widely investigated and shown to elicit benefits to performance in different tasks (e.g., endurance running, explosive throws, etc.) (McGowan et al. 2015; Seitz and Haff 2016). In addition, specially designed clothing is proposed to acutely enhance sports performance. More specifically, a systematic review suggested that compression garments are capable of improving sprinting and jumping performances, but with small effect sizes (ES) (Born et al. 2013). However, results in the literature are mixed (MacRae et al. 2011), and in some instances do not relate to athletic performance, besides not addressing the meaningfulness of the results (using specially-featured statistics) to actual sports settings.

In one of the first studies on this topic, Kraemer et al. (1996) showed that volleyball players were better able to maintain power output during a repeated jump test while wearing compression shorts compared to control garments (regular gym shorts); however, maximal jump power in the best attempt was not altered by the compression. Compression garments did not demonstrate benefits in 20-m sprint or countermovement jump performances in netballers (Higgins et al. 2009). However, the use of upper body compression garments resulted in improved accuracy in both baseball pitching and golf shots (Hooper et al. 2015). Positive changes in performance could be at least partly attributed to improved proprioception, which is the awareness of body segments and position in space, by means of greater sensory feedback due to the compressive effects of the clothing (Born et al. 2013).

Supposedly, Paralympic athletes with visual impairment rely more on proprioception to perform in their respective sports than their Olympic peers. Therefore, it can be hypothesized that sprinters with visual impairment will present benefits from the use of lower limb compression garments while performing speed–power tests. The aim of this study was to explore the effects of using compression garments on unloaded and loaded vertical jump and sprinting performances, in elite sprinters with visual impairment.

## Methods

### Participants

Eight top-level Paralympic sprinters with visual impairment (6 women and 2 men; class T11) (age: 27.8 ± 6.7 years, weight: 62.2 ± 11.2 kg, and height: 165.9 ± 10.0 cm) who compete in 100- and 200-m races, from the Brazilian National Team, volunteered to participate in this study. This elite sample comprised two world champions, two Paralympic champions, one world record holder, one Paralympic record holder, seven world medalists and four Paralympic medalists. In addition, all the eight athletes are top-five in the 2015 International Paralympic Committee (IPC) world ranking, thus attesting their high level of competitiveness. The procedures were approved by an Institutional Ethics Committee. After being fully informed of the risks and benefits associated with the study, all athletes signed a written informed consent form.

### Study design

**Fig. 1:**
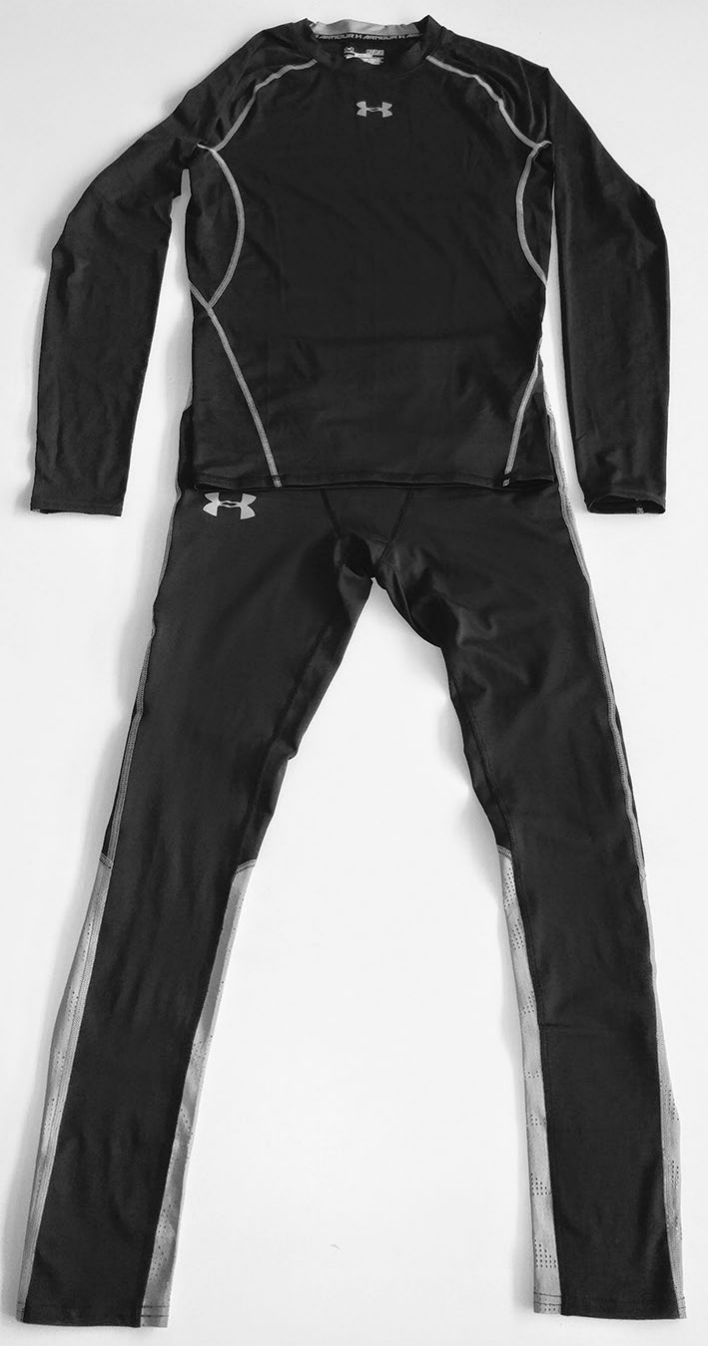
Compression garment worn by the Paralympic athletes

This is a pilot study using a within-subject randomized cross-over experimental design to test the effectiveness of compression garments on speed–power tests performance in eight top-level Paralympic sprinters. Four of them wore the compression garments on the first testing day, while the other four athletes wore the non-compressive clothes (“control condition”). The “control condition” consisted of wearing a non-compressive Lycra^®^ clothing, whereas the “compression condition” consisted of using garments with a functional compressive-body composed of 84 % nylon and 16 % elastane (Under Armour, Baltimore, MD, USA). The athletes performed, in the following order, unloaded squat jump (SJ), loaded jump squat (JS) and a sprint test over 20- and 70-m distances; using or not the lower body and upper body compression garment (Fig. 1). The tests were performed on the same day, with 5–10 min separating each test. Prior to the two testing sessions, the participants dressed the assigned clothes and executed a standardized warm-up protocol, including general (i.e., running at a moderate pace for 10-min followed by 5-min of active lower limb stretching) and specific exercises (i.e., sprint drills and low-intensity plyometrics). The warm-up was followed by a 3-min rest interval, after which the athletes were required to perform the actual tests. The test days were interspersed with 48-h, a period during which the athletes were oriented not to heavily train and to maintain their habitual dietary habits. In the 24-h prior to testing, the athletes were also requested not to consume alcohol or caffeine-based beverages. The study was conducted during the last semester of the final preparation phase of the cycle leading up to the 2016 Paralympic Games.

### Squat jump (SJ) test

Unloaded vertical jumping ability was assessed using SJ, which started from a static position with a 90° knee flexion angle maintained for 2-s prior to a maximal jump attempt without any preparatory movement. Jumps were executed with the hands on the hips, and visually validated by one of the investigators, making sure that take-off and landing in the vertical jumps were performed at the same lower-limb position (extended knees). Otherwise, the given jump was repeated. The jumps were performed on a contact platform (Smart Jump; Fusion Sport, Coopers Plains, Australia) with the obtained flight time (t) being used to estimate the height of the rise of the body’s center of gravity (h) during the vertical jump (i.e., h = gt^2^/8, where g = 9.81 m s^−2^). Five attempts were performed interspersed with 15-s intervals. The best attempt was used for data analysis purposes. The athletes executed the SJ attempts without any help from their guides.

### Mean propulsive power in loaded jump squat (JS)

Mean propulsive power was assessed using the jump squat exercise (MPP JS), performed on a Smith machine (Hammer Strength, Rosemont, IL, USA). The Paralympic sprinters executed a knee flexion until the thigh was parallel to the ground (≈100° knee angle) and, following a command, jumped as fast as possible, without their shoulder losing contact with the bar. The athletes were instructed to execute two repetitions at maximal velocity for each load, starting at 40 % of their body mass (BM), after which a load of 10 % of BM was gradually added in each set until a decrease in MPP was observed. A 5-min interval was provided between sets. To determine mean propulsive power, a linear transducer (T-Force, Dynamic Measurement System; Ergotech Consulting S.L., Murcia, Spain) was attached to the Smith machine bar. The technical specification of the MPP analysis and calculation, and the use of the MPP rather than peak power have been described previously (Loturco et al. 2015a; Sanchez-Medina et al. 2010). The maximum MPP value obtained was considered for data analysis purposes. The athletes executed the SJ attempts without any help from their guides.

### Sprinting speed

Prior to the execution of the sprint speed test, three pairs of photocells (Smart Speed, Fusion Equipment, Australia) were positioned at distances of 0-, 20- and 70-m along the indoor track and field course. The Paralympic athletes sprinted twice, starting from a standing position, 0.3-m behind the start line. A 5-min interval was allowed between the two attempts and the fastest time obtained was considered for further analyses. The sprinters performed the sprint tests accompanied by their official guides.

### Statistical analysis

Data are presented as mean ± SD. The comparisons of the test performances between the two conditions (placebo and compression) were analyzed using magnitude-based inference (Batterham and Hopkins 2006). The quantitative chances of finding differences in the variables tested were assessed qualitatively as follows: <1 %, almost certainly not; 1–5 %, very unlikely; 5–25 %, unlikely; 25–75 %, possible; 75–95 %, likely; 95–99 %, very likely; >99 %, almost certain. If the chances of having better and poorer results were both >5 %, the true difference was assessed as unclear. The standardized differences for the comparisons in all variables were analyzed using the Cohen’s *d* standardized differences based on ES (Cohen 1988). The magnitudes of the ES were qualitatively interpreted using the following thresholds: <0.2, trivial; 0.2–0.6, small; 0.6–1.2, moderate; 1.2–2.0, large; 2.0–4.0, very large and; >4.0, nearly perfect (Hopkins 2004; Hopkins et al. 2009).

## Results

**Fig. 2:**
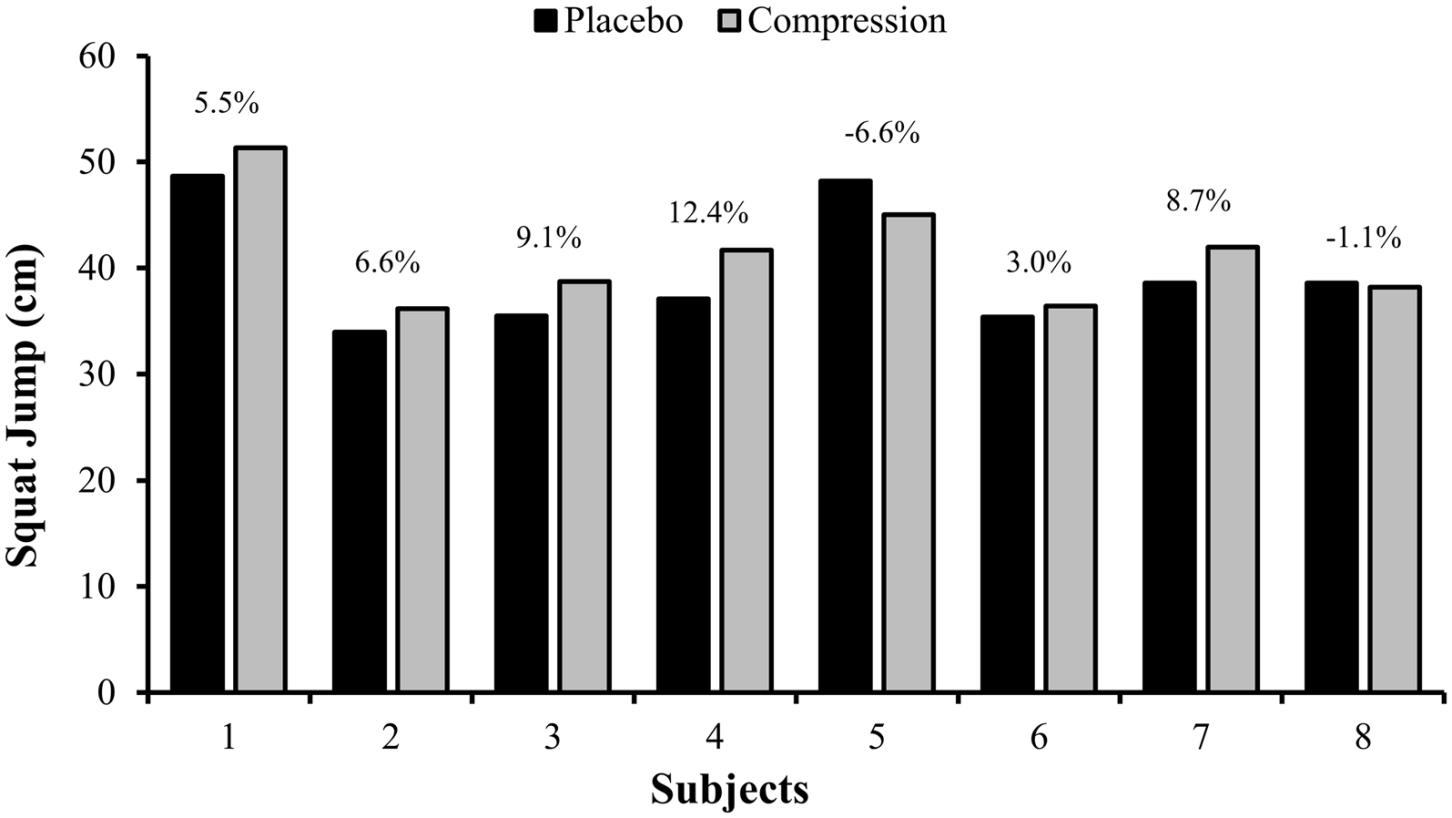
Individual performance differences between the placebo and compression conditions in the squat jump (SJ) exercise

Table 1 shows the comparisons between the unloaded (SJ) and loaded (MPP JS) vertical jumps and 20- and 70-m sprint performances in the placebo and compression conditions. The SJ was *possibly higher* in the compression than in the placebo condition (placebo: 39.49 ± 5.75 cm; compression: 41.19 ± 5.09 cm). The difference between the placebo and compression conditions in the MPP JS was rated as *unclear* (placebo: 484.06 ± 158.20 W; compression: 474.24 ± 147.70 W). Finally, the differences in the 20- and 70-m sprint times between the placebo and compression were all rated as *unclear* (placebo: 3.24 ± 0.20 s; compression: 3.27 ± 0.11 s, for 20-m; and placebo: 9.12 ± 0.44 s; compression: 9.07 ± 0.39 s, for 70-m). Figure 2 displays the individual performances in the placebo and compression conditions for the SJ.

**Table 1:** Comparisons between the unloaded (SJ) and loaded (MPP JS) vertical jumps and 20- and 70-m sprint performances in the placebo and compression conditions

	Placebo	Compression	Effect size (90 % CI)	Qualitative inference
SJ (cm)	39.49 ± 5.75	41.19 ± 5.09	0.26 (0.01; 0.52)	*Possibly*
MPP JS (W)	484.06 ± 158.20	474.24 ± 147.70	−0.06 (−0.18; 0.07)	*Unclear*
20 m sprint (s)	3.24 ± 0.20	3.27 ± 0.11	0.15 (−0.54; 0.83)	*Unclear*
70 m sprint (s)	9.12 ± 0.44	9.07 ± 0.39	−0.10 (−0.42; 0.23)	*Unclear*

*CI* confidence interval, *SJ* squat jump, *MPP JS* mean propulsive power in the jump squat exercise

## Discussion

This is the first study to test the effects of compression clothing on the speed and power related abilities of Paralympic sprinters. The main finding reported herein is that, in this highly selected group of elite athletes with visual impairment, the compression garments were able to induce acute improvements in the unloaded vertical jumping ability, as assessed by the increases in the SJ height. Since SJ performance is strongly associated with competitive results in Paralympic sprint events (Loturco et al. 2015c), this finding may have important implications in the field of sport science.

In fact, a previous research involving elite sprinters with visual impairment has shown that the smallest worthwhile enhancements in 100- and 200-m competitive results could be detected by the smallest worthwhile enhancements in the SJ height (Loturco et al. 2015c). Remarkably, this study was conducted throughout a training cycle composed of seven different official competitions (four national, two international and the Parapan American Games 2015), which strengthens the practical relevance of these findings (Loturco et al. 2015c). Furthermore, a pooled correlational analysis of the data collected in five different testing sessions and the actual performance achieved by these Paralympic athletes in the corresponding competitions revealed a very close relationship (r ≈ 0.80) between 100- and 200-m sprint times and SJ height. Although it is recognized that correlations do not necessarily imply causality, it is reasonable to consider that an “optimized jumping condition”—as caused by the use of compression garments—might induce positive adaptations in maximal sprinting performance.

The mechanical and biological reasons behind the acute enhancements caused by compression clothing on the unloaded vertical jumping performance of athletes with visual impairments remain speculative and require further research. However, a conceivable explanation for this phenomenon might be related to the well-established positive effects of compression garments on proprioceptive cues (Hooper et al. 2015; Kraemer et al. 1996). This possibly affects the performance of blind athletes, who rely on high levels of proprioception to successfully execute their specific motor tasks (Grace Gaerlan et al. 2012; Pereira et al. 2016). In these individuals, the absence of regular visual feedback probably disturbs the dynamic postural balance (Grace Gaerlan et al. 2012), which may hamper their performance in vertical jumps. Indeed, it seems that compressive forces can interact with biological cueing systems, eliciting enhancements in joint proprioception and the ability to perceive position in space (Kraemer et al. 1996, 1998). Furthermore, when used in clinical settings, compression garments have revealed their essential biomechanical properties, by demonstrating a positive “dampening effect”, capable of reducing overshooting of the limbs and increasing mechanical stability during some specific motor tasks (which is recognized as a determinant factor in vertical jump performance) (Butcher et al. 2007; Hibbs et al. 2008; Hylton and Allen 1997; Pearce et al. 2009). Importantly, in our study, the Paralympic athletes simultaneously wore compression clothing on the upper and lower limbs, which most likely produced an amplified effect in these performance-enhancing capacities.

Curiously, these “neuromechanical effects” were not able to improve the muscle power production in the loaded JS exercise. Similarly, Kraemer et al. (1998) reported that compression garments did not augment the maximal power output for the highest vertical jump during a sequential fatigue test (although enhanced mean power production was observed throughout the consecutive jumps). For the authors, this lack of improvement in maximal muscle power production may be partially related to the level of compression applied by the garment. In fact, it has already been reported that higher levels of compression applied around the knees can substantially increase the vertical force component during competitive lifts, and therefore augment maximum strength performance in the squat exercise (Harman and Frykman 1990). Actually, in this study, we opted to use comfortable compression clothing (with moderate levels of compression), since the subjects had to perform a considerable series of tests wearing these garments. It is possible that this strategy markedly affected our outcomes, producing positive changes only in unloaded jumps, which are executed at higher velocities. From a mechanical perspective, to improve the performance in vertical loaded jumps (which depend on higher levels of force applied at lower velocities), it is probably necessary to adopt garments with extreme levels of compression (e.g., undersized garments). More importantly, these vestments should be designed to apply higher levels of compression around the joints involved in the assessed movement. However, this assertion needs to be confirmed or rejected by means of experimental studies comparing garments with different intensities/locations of compression.

The absence of positive effects on the maximal sprinting speed performance is not a novelty in the context of compressive clothing. In agreement with our results, Faulkner et al. (2013) reported that compression garments were not able to improve the overall 400-m sprint performance or individual 100-m split times in elite sprinters. On the other hand, the authors demonstrated that these clothes might lower the effort perception associated with long-sprint performance (i.e., 400-m). Although we performed our speed tests over shorter distances (20- and 70-m), we also could not detect any important variation in the actual sprint times of the Paralympic athletes under either experimental condition (i.e., compression or placebo). To some extent, the agreement between studies’ results (despite the differences in the athletic population) might be attributed to the high correlation between performances in short (35-m) and long (400-m) sprints (Paradisis et al. 2005), meaning that sprint ability over several distances is determined by similar neuromechanical factors. It seems that, even with their pronounced proprioceptive qualities, blind athletes do not demonstrate substantial improvements in their maximal sprinting capacity when using compression clothing. Possibly, the “multifactorial nature” of sprint performance (Cronin and Hansen 2005; Loturco et al. 2015b) limits the role played by comfortable levels of compression in increasing maximal speed, especially in top-level athletes. Nevertheless, due to the recognized properties of compression in reducing the perception of effort and facilitating blood lactate removal (Faulkner et al. 2013), it would be interesting to test its effectiveness during repeated-sprint sets. Furthermore, it remains to be examined whether garments with extreme levels of compression can enhance top-speed performance, since it was demonstrated that very-high intensity compression may increase the vertical force component (which is directly related to higher velocities) (Harman and Frykman 1990; Nilsson and Thorstensson 1989; Weyand et al. 2000).

This study is limited by a number of factors, such as the absence of a control group, the small sample size and the lack of physiological and psychological additional measures (e.g., hemodynamics and thermal comfort). However, this investigation was performed with eight of the best top-level Paralympic sprinters in the world, who together have already accumulated more than ten international titles, among them the World Championships, Paralympic Championships and Pan-American Games. Therefore, even considering the *possibly small* positive effects of compression clothing on the unloaded jumping performance (4.7 %, on average), this information might be extremely valuable for Paralympic coaches, since minimal differences in sprint times represent an enormous gap in top-level Paralympic sprint performance (Loturco et al. 2015c). It is important to emphasize that, for this group of elite athletes with visual impairment, the variation in the unloaded SJ height is strongly related to the variation in the actual competitive performance in 100- and 200-m dash events. Additionally, for top-level sprinters, variations of about 1 % in real race times can be considered meaningful (Hopkins 2004; Loturco et al. 2015c; Pereira et al. 2016), based on statistical concepts specially formulated to assess Olympic and Paralympic athletes.

## Conclusions

This pilot investigation opens new perspectives to test the effectiveness of compression clothing on Paralympic sports performance. Supported by these preliminary findings, Paralympic athletes with visual impairment are encouraged to wear compression garments during their power-related training sessions, especially when attempting to optimize muscle power outputs under unloaded situations (e.g., plyometrics). In these circumstances, the possibility of increasing the muscle power production by using these garments is probably related to their properties of enhancing proprioceptive cues (which play a determinant role in Paralympic sports performance). Further studies should be conducted to test the efficiency of these clothes on optimizing performance during repeated-sprint sets, during which the recognized recovery-enhancement properties of the clothes could also be explored. Finally, experimental trials employing clothes with extreme levels of compression are highly desired, since it is expected that these garments positively influence vertical force production and, therefore, actual top-speed performance.
